# Site-Specific Variations in Bone Mineral Density under Systemic Conditions Inducing Osteoporosis in Minipigs

**DOI:** 10.3389/fphys.2017.00426

**Published:** 2017-06-20

**Authors:** Matthias C. Schulz, Jan Kowald, Sven Estenfelder, Roland Jung, Eberhard Kuhlisch, Uwe Eckelt, Ronald Mai, Lorenz C. Hofbauer, Christian Stroszczynski, Bernd Stadlinger

**Affiliations:** ^1^Department of Oral and Maxillofacial Surgery, Medical Faculty “Carl Gustav Carus,” Technische Universität DresdenDresden, Germany; ^2^Department of Radiology, Medical Faculty “Carl Gustav Carus,” Technische Universität DresdenDresden, Germany; ^3^Division of Nephrology, Department of Internal Medicine III, Medical Faculty “Carl Gustav Carus,” Technische Universität DresdenDresden, Germany; ^4^Department of Internal Medicine III, University of UlmUlm, Germany; ^5^Experimental Center, Medical Faculty “Carl Gustav Carus,” Technische Universität DresdenDresden, Germany; ^6^Institute for Medical Informatics and Biometry, Medical Faculty “Carl Gustav Carus,” Technische Universität DresdenDresden, Germany; ^7^Division of Endocrinology, Diabetes and Bone Diseases, Department of Medicine III, Medical Faculty “Carl Gustav Carus,” Technische Universität DresdenDresden, Germany; ^8^Department of Radiology, University Hospital RegensburgRegensburg, Germany; ^9^Clinic of Cranio-Maxillofacial and Oral Surgery, University of Zurich, University Hospital ZurichZurich, Switzerland

**Keywords:** animal models, bone, bone mineralization, oral cavity, osteoporosis

## Abstract

Osteoporosis is a systemic bone disease with an increasing prevalence in the elderly population. There is conflicting opinion about whether osteoporosis affects the alveolar bone of the jaws and whether it poses a risk to the osseointegration of dental implants. The aim of the present study was to evaluate the effects of systemic glucocorticoid administration on the jaw bone density of minipigs. Thirty-seven adult female minipigs were randomly divided into two groups. Quantitative computed tomography (QCT) was used to assess bone mineral density BMD of the lumbar spine as well as the mandible and maxilla, and blood was drawn. One group of minipigs initially received 1.0 mg prednisolone per kg body weight daily for 2 months. The dose was tapered to 0.5 mg per kg body weight per day thereafter. The animals in the other group served as controls and received placebo. QCT and blood analysis were repeated after 6 and 9 months. BMD was compared between the two groups by measuring Hounsfield units, and serum levels of several bone metabolic markers were also assessed. A decrease in BMD was observed in the jaws from baseline to 9 months. This was more pronounced in the prednisolone group. Statistically significant differences were reached for the mandible (*p* < 0.001) and the maxilla (*p* < 0.001). The administration of glucocorticoids reduced the BMD in the jaws of minipigs. The described model shows promise in the evaluation of osseointegration of dental implants in bone that is compromised by osteoporosis.

## Introduction

Osteoporosis is a systemic disease that affects bone mass and architecture, increasing the risk of bone fractures (Rachner et al., 2011). Osteoporosis is prevalent in the United States, Europe, and Japan, causing over 4.5 million fractures annually (WHO Scientific Group on the Assessment of Osteoporosis at Primary Health Care Level, 2004). As consequence of an aging population, an increasing number of people suffer from osteoporosis. The most common form of osteoporosis is post-menopausal osteoporosis in females, due to reduced estrogen levels. Other risk factors for the development of osteoporosis include patient age and long-term systemic exposure to exogenous glucocorticoids. The clinical diagnosis of osteoporosis is usually confirmed radiographically, via dual-energy X-ray absorptiometry (DXA)-based bone densitometry (Felsenberg and Gowin, 1999) and, more recently, with high resolution quantitative computed tomography (hrQCT; Snedeker et al., 2006; Graeff et al., 2013).

The influence of both local and systemic diseases on the osseointegration of dental implants has been a repeated point of discussion. For example, it has been shown that periodontitis in conjunction with smoking (Heitz-Mayfield and Huynh-Ba, 2009), and radiotherapy prior to implant placement (Chambrone et al., 2013), negatively affect implant integration. With regard to osteoporosis, there are contradictory reports on the success rates of dental implants placed in osteoporotic bone. Whereas, some studies describe no influence of osteoporosis on the implant osseointegration (Dvorak et al., 2011a), other authors report higher loss rates of implants in osteoporotic bone (Moy et al., 2005; Alsaadi et al., 2008). It is uncertain whether osteoporosis commonly induces areas of rarefaction in the alveolar processes of the jaws that are of clinical relevance to implant success. While some authors report that osteoporotic skeletal changes are reflected in alveolar bone changes (Jonasson et al., 2006), others have found no correlation between the condition of the general skeletal and alveolar bone structures (Kingsmill and Boyde, 1999).

Such possible correlations can be analyzed in animal models. In osteoporosis research, rodent models have been used for analysis of the integration of biomaterials (Stadlinger et al., 2013). In rodent studies, ovariectomy serves to induce an osteoporotic state. Large animal models more closely resemble human anatomy and human patterns of wound healing, allowing the application of regular-sized surgical instruments and dental implants. However, due to practicalities, it has been uncommon to employ systemically impaired large animal models for the analysis of dental implant integration in bone or soft tissue (Thoma et al., 2012). Sheep, minipigs, and dogs may serve for such studies. The establishment of an osteoporotic state in large animals is possible by various means. Usually osteoporosis is induced by ovariectomy, however this has been reported to have a limited influence on nulliparous pigs (Scholz-Ahrens et al., 1996). Other approaches involve a calcium-deficient diet, the systemic application of glucocorticoids, or a combination of those approaches. Recently, an animal model has been reported, showing mandibular bone loss following a hypothalamic-pituitary disconnection (Oheim et al., 2014).

The application of glucocorticoids in minipigs has been shown to induce reduction of BMD in the lumbar spine as assessed by quantitative computed tomography (QCT; Scholz-Ahrens et al., 2007). QCT enables repeated measurements of the entire skeleton over time and is therefore suitable for large animal studies on bone structure.

In most pre-clinical studies, implant integration is analyzed in pristine bone under unimpaired osseous conditions. The aim of this minipig study was to establish a large animal model with glucocorticoid-induced osteoporosis, for the analysis of implant osseointegration under impaired systemic osseous conditions. To achieve this effect, the experimental animals received systemic glucocorticoid therapy for 9 months. During this period, the animals underwent three QCT assessments. The hypothesis was that glucocorticoid application for 9 months would induce a significant decrease of BMD in both the lumbar spine and the alveolar bone of the jaws.

## Materials and methods

### Experimental design

The study protocol was approved by the Commission for Animal Studies at the District Government, Dresden, Germany (file reference: 24-9168.11-1/2008-43). The study was conducted in accordance to the recommendations and guidelines of the Commission for Animal Studies at the District Government, Dresden, Germany.

For the study, 37 adult female miniature pigs (nulliparous, aged 18–20 months, average weight 72.5 ± 16.7 kg) of the breed Mini-Lewe were used. The animals were randomly divided into two groups: Group I (29 animals) received daily oral prednisolone, 1 mg per kg body weight in the initial phase. After 2 months, the dosage was reduced to 0.5 mg prednisolone per kg body weight per day. Prednisolone administration was performed according to the protocol described by Scholz-Ahrens (Scholz-Ahrens et al., 2007). Group II (eight animals), which received neither prednisolone nor a carrier, served as control. Before the commencement of prednisolone, and at the 6- and 9-month time-points of prednisolone administration, QCT was performed for each animal, and blood samples for laboratory assessment and measurement of biochemical markers of bone turnover were also taken.

### Nutrition

Prior to the start of the study, the animals were fed a diet consisting of pollard, wheat bran, and corn silage obtained from a local food producer. The pollard contained 0.57% of calcium and 0.55% of phosphorus. The content of vitamin D3 was 328 international units (IU) per kg. After initiation of the glucocorticoid therapy, the experimental animals were fed a calcium-deficient diet omitting the pollard. The diet contained 58.3% wheat bran and 41.7% corn-based silage. The wheat bran contained 0.07% calcium and 0.05% phosphorus. The content of vitamin D3 was <300 IU per kg. The diet of the control group remained unchanged. Water was available *ad libitum*. The components of the animal nutrition were analyzed prior to the study (LUFA-ITL GmbH, Kiel, Germany).

### Sedation

Computed tomography investigations and the collection of the blood samples were performed under sedation, under the surveillance of a veterinarian. The medication for sedation involved a mixture of 1 mg/kg body weight midazolam (Ratiopharm GmbH, Ulm, Germany), 10 mg/kg body weight ketamine (Riemser Arzneimittel AG, Greifswald, Germany), and 0.05 mg/kg body weight atropine (Eifelfango Chem.-Pharm. Werke, Bad Neuenahr-Ahrweiler, Germany), administered intramuscularly.

### Quantitative computed tomography

The computed tomography scanner was a multislice-CT (SOMATOM 16, Siemens Healthcare, Erlangen, Germany). The minipigs were positioned ventrally due to veterinary aspects of respiration, with mandible and maxilla parallel to the horizontal plane. The images were acquired by applying the spiral technique with a collimation of 16 × 0.75 mm. The data acquisition was performed axial using a slice-thickness of 0.75 mm. The field of view was chosen 265 × 265 mm for the jaw scan and 200 × 200 mm for the scan of the lumbar vertebrae with a matrix of 512 × 512. Thus, a pixel spacing of 0.426 × 0.426 mm for the jaw scan and of 0.445 × 0.445 mm resulted. The optimal set-up conditions for the jaw scan were a tube voltage of 120 kV and 36 mAs. For the vertebra region the same tube voltage was used with 274 mAs.

The data was acquired by a filtered back projection with convolution kernel H60s for the jaw scan and B70s for the vertebra scan. Saving was performed as digital imaging and communication in medicine (DICOM) standard using the Leonardo workstation (LEONARDO VD10B; Syngo VX49B; Siemens Healthcare Diagnostics GmbH, Eschborn, Germany). The analyses and measurements of the CT scans were performed with interactive data language software (IDL 7.0.0, ITT-Visual Information Solutions, Boulder/CO, USA) by two experienced examiners (JK, SE). To calculate the inter- and intra-observer reproducibility, measurements of eight randomly selected animals were repeated twice by each examiner with a time interval of 2 weeks. Calibration was performed using a reference phantom containing a bone-like and a water-like phase (Osteo Phantom, Siemens Healthcare, Erlangen, Germany) placed parallelly to the horizontal plane. A gray scale of Hounsfield units (HU) served for the analysis of the bone mineral density. A threshold was defined in order to ensure that no soft tissue (e.g., fat) or dental structures (e.g., roots) were included in the measurements. In preliminary tests, a mean density of the mandibular and maxillary cortical bone of 1386 HU was assessed (data not shown). Furthermore, the gray values of the soft tissues in the bone marrow were measured to have a peak value below 0 HU. According to the resulting histograms, the thresholds at 0 HU for the minimum and at 1,350 HU for the maximum were defined. As mineralized dental structures show comparable or higher density values than cortical bone they could be precluded from the measurements by the threshold, likewise.

**Figure 1:**
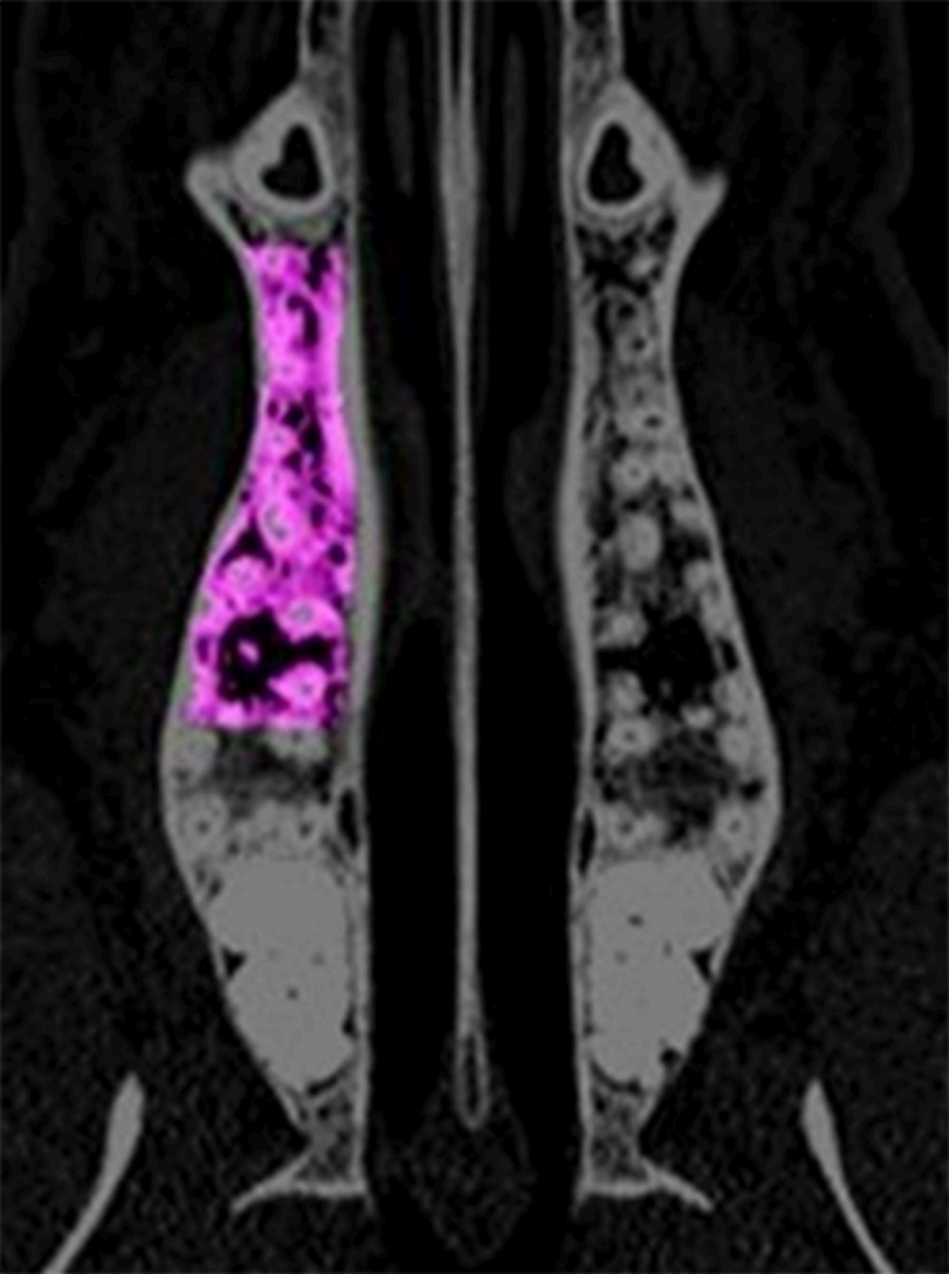
The region of interest for the analysis of the computed tomography scans is depicted in the maxilla. The area is extended between the distal edge of the canine root and the mesial edge of the third molar. Laterally, the cortical bone served as border.

**Figure 2:**
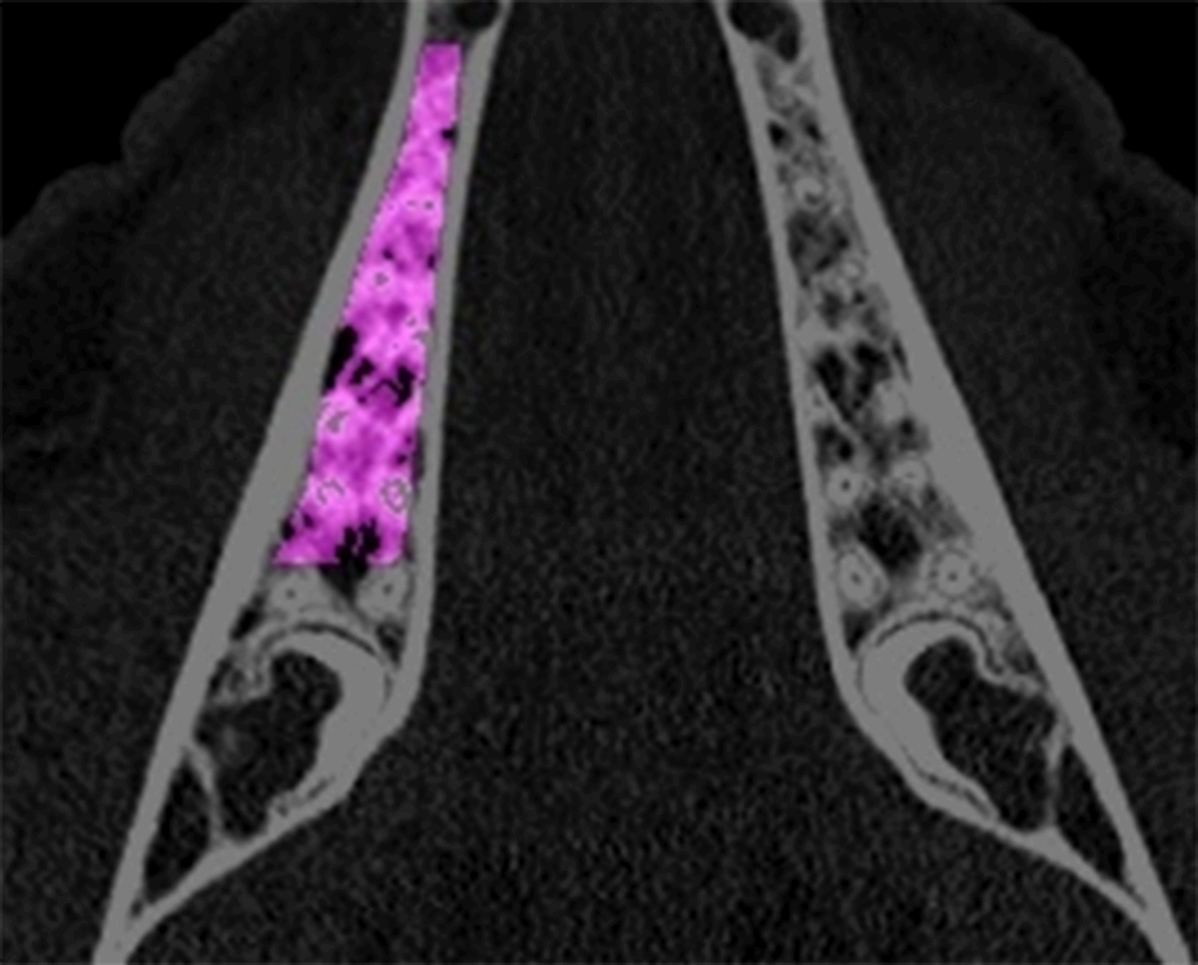
The region of interest for the analysis of the computed tomography scans is depicted in the mandible. The area is extended between the distal edge of the canine root and the mesial edge of the third molar. Laterally, the cortical bone served as border.

**Figure 3:**
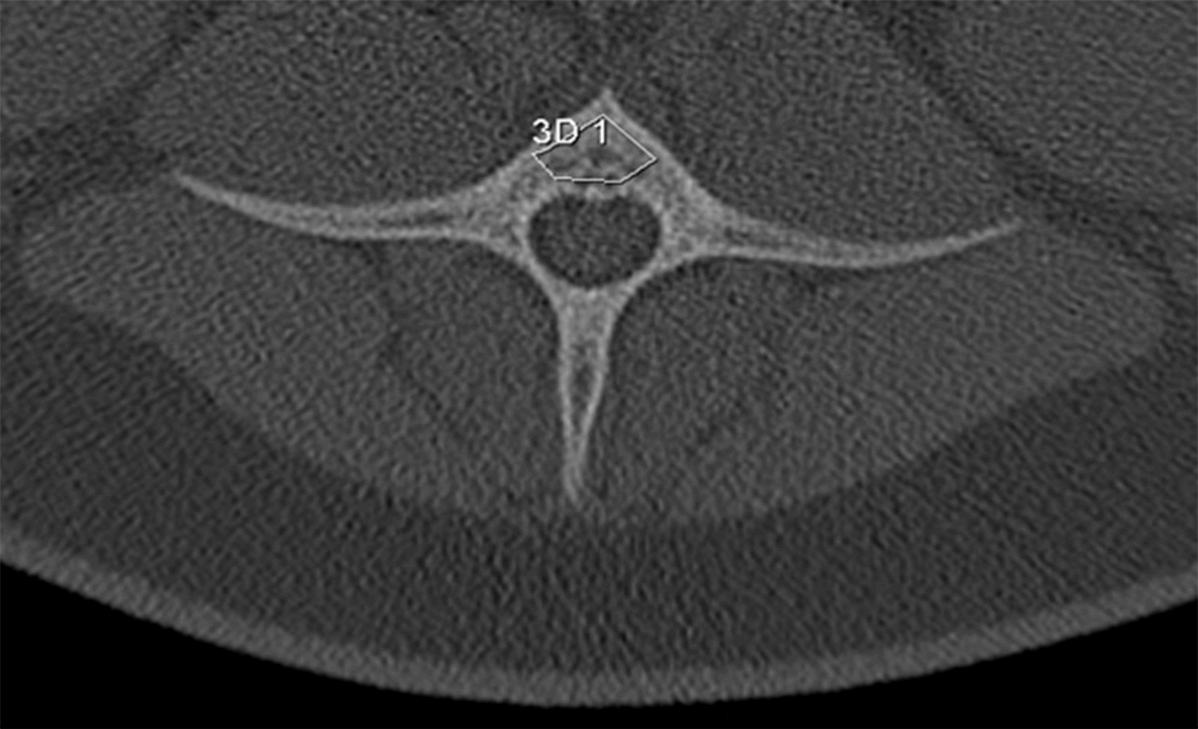
In the vertebrae, the region of interest was defined as a polygon being limited by the ventral and dorsal cortical bone and dorso-laterally by a dense trabecula between the ventral cortical bone and the spinal canal.

For the radiographic analysis of the jaw region, the radiographs were positioned parallelly to the transversal plane. The vertical position of the transversal cutting plane was determined by the root tips of the three premolar teeth in each jaw. The regions of interest in the mandible and maxilla were defined as follows: The oral and buccal borders were defined by the inner layer of the cortical bone. The distal edge of the canine root served as the anterior border and the mesial root of the third molar as the distal border. The matching of the radiographs of the baseline QCT and of the QCTs after 6 and 9 months was achieved by using constant anatomical structures e.g., pterygoid process, the bony nasal septum and the tuber maxilla in the upper jaw. In the mandible, the profile of the oral and vestibular cortical bone was used for the matching of the images. For the measurement of the BMD in lumbar vertebrae I–III, the radiographs were positioned parallel to the axial plane. The region of interest was defined through the ventral cortical bone and the cortical bone of the spinal canal. The lateral border was defined by a dense trabecula connecting the ventral cortical bone and the spinal canal in dorsomedial direction. The regions of interest are depicted in Figures 1, 2 for the jaw region and in Figure 3 for the vertebral region. For the measurement of the bone density, the gray values of the pixels in the regions of interest were assessed and displayed in Hounsfield Units using the IDL-software. The mean value and standard deviation of each region of interest was calculated. In the upper and lower jaw, the left and right side were measured separately, and were later pooled for the statistical evaluation.

### Serum parameters

Blood samples were taken before the commencement of glucocorticoid administration and after 6 and 9 months, respectively. The samples were always obtained from the ear vein at the same time of the day using serum gel monovettes (Sarstedt AG & Co., Nürmbrecht, Germany) and stored at 4°C until centrifugation. Subsequently, the following parameters were analyzed using an automatic test system (cobas c, Roche Diagnostics, Mannheim, Germany). The serum level of ionic calcium and ionic phosphorus were analyzed by photometric test kits (CA2 and PHOS2). The level of total alkaline phosphatase was measured by an extinction test kit (ALP2). For the analysis of the levels of beta crosslaps an immunoassay test kit (ECLIA) was used. All test kits were obtained from Roche Diagnostics, Mannheim, Germany. Bone specific alkaline phosphatase was measured using an immunoassay (MicroVue™ BAP, Quidel Corporation, San Diego, CA, USA). The serum level of 25(OH) Vitamin D was analyzed by a chemiluminescence assay (LIAISON® 25 OH Vitamin D TOTAL Assay). The analysis of the osteocalcin level was performed using a two-site immunoradiometric assay (CA 72-4 IRMA). Both assays were obtained from DiaSorin Inc., Stillwater, MN, USA. Osteoprotegrin (OPG) levels were analyzed by an enzyme-linked immune sorbent assay (Osteoprotegrin ELISA kit, Immundiagnostik AG, Bensheim, Germany). The serum level of 1,25(OH)_2_-Vitamin D was measured using a radioimmunoassay (1,25-Dihydroxy Vitamin D RIA, Immunodiagnostic Systems GmbH, Frankfurt am Main, Germany). All analyses were performed by the Institute for Clinical Chemistry and Laboratory Medicine (Medical Faculty “Carl Gustav Carus,” Technische Universität Dresden, Dresden, Germany) using standardized laboratory methods.

### Statistical analysis

The degree of agreement between the two observers and the degree of agreement between the two measurements were assessed by a reliability analysis. The inter- and intra-observer reliability is described by the intraclass correlation coefficient (ICC) taking a two-way random model with absolute agreement definition. The animal experiment was planned and carried out as longitudinal study. Consequentially the analyses were done by ANOVA using linear mixed models. The models included fixed effects associated with treatment, time, and the interaction between treatment and time. The residual covariance matrix was specified as a heterogeneous compound symmetry (CSH), so that the variances differ across the levels of time. Estimated means and their 95%-confidence limits are given. *Post-hoc* tests and confidence limits are alpha-adjusted using the Tukey–Kramer method. The statistical analysis was performed using SPSS 19 (SPSS Inc., Chicago, Illinois, USA) and SAS 9.3 (SAS Institute Inc., Cary, NC, USA).

## Results

From the initially 37 minipigs, two animals of the prednisolone group died due to unknown cause and were excluded from the analysis. Thus, 35 animals completed the study period and could be included in the evaluation.

### Quantitative computed tomography

#### Intra- and inter-observer control

The intra-observer analysis showed an ICC of 0.992 (95%-confidence interval: 0.987–0.995) for observer I and an ICC of 0.991 (95%-confidence interval: 0.987–0.994) for observer II. The inter-observer analysis showed an ICC of 0.992 (95%-confidence interval: 0.988–0.995) for the first time point and an ICC of 0.990 (95%-confidence interval: 0.984–0.993) for the second time point. A statistically significant difference between both observers could not be found, neither for the first (*p* = 0.592) nor for the second time point (*p* = 0.224).

#### Control group—development of bone mass with aging

With regard to BMD-values, the control group showed a general decrease from baseline to the 6-month examination. This decrease was statistically significant for all regions of interest. From 6 to 9 months, a statistically significant increase of BMD was obvious for all examined regions. When comparing the baseline values to those obtained after 9 months, an overall decrease of BMD was found. However, this decrease was only statistically significant for the maxilla. For vertebrae and mandible no significant changes could be found (vertebrae: *p* = 0.988; mandible: *p* = 0.995).

#### Effects of glucocorticoid exposure on bone mass

**Figure 4:**
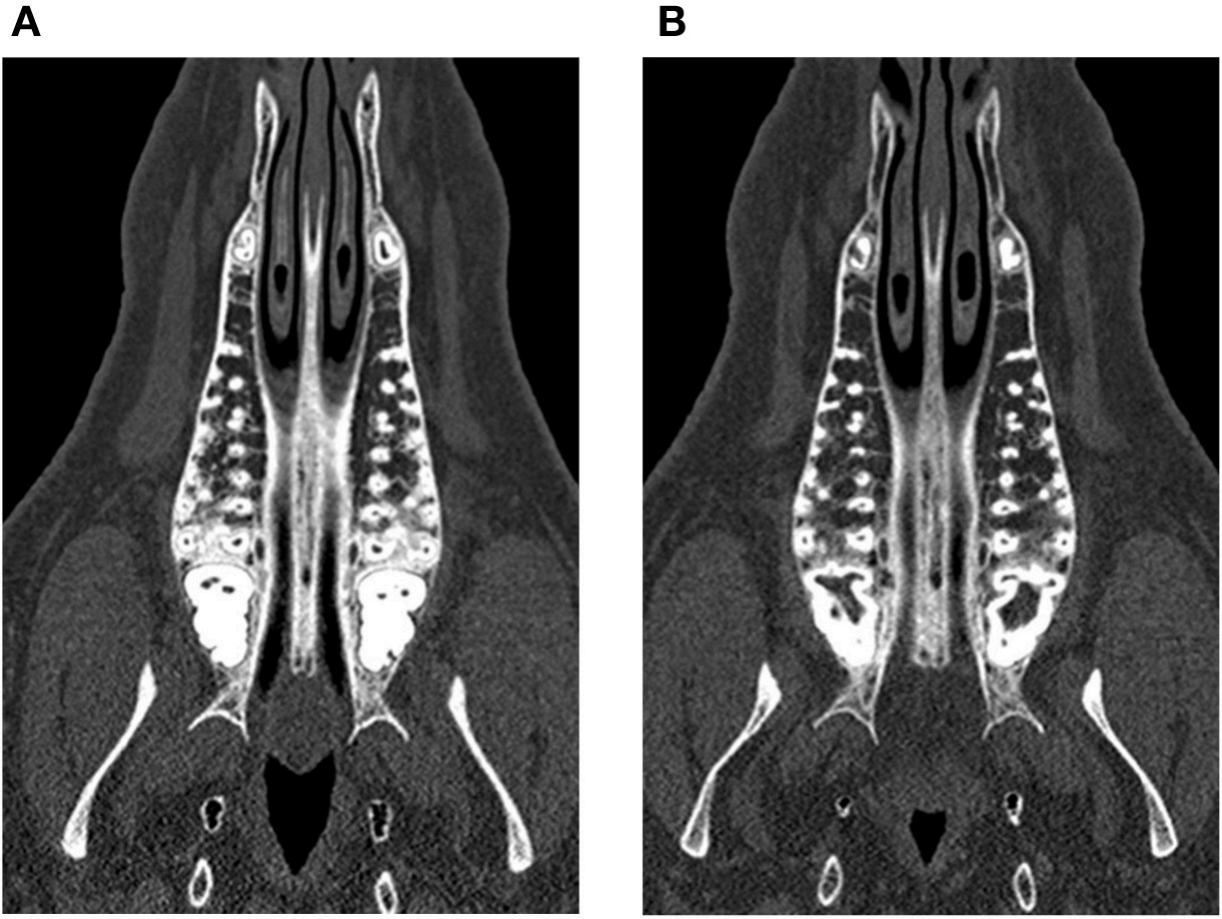
Trabecular rarefication over time shown in the maxilla. Compared to the baseline **(A)** the spongious area in the region of interest appears in darker gray values after 9 months **(B)** indicating a loss of mineralization.

**Figure 5:**
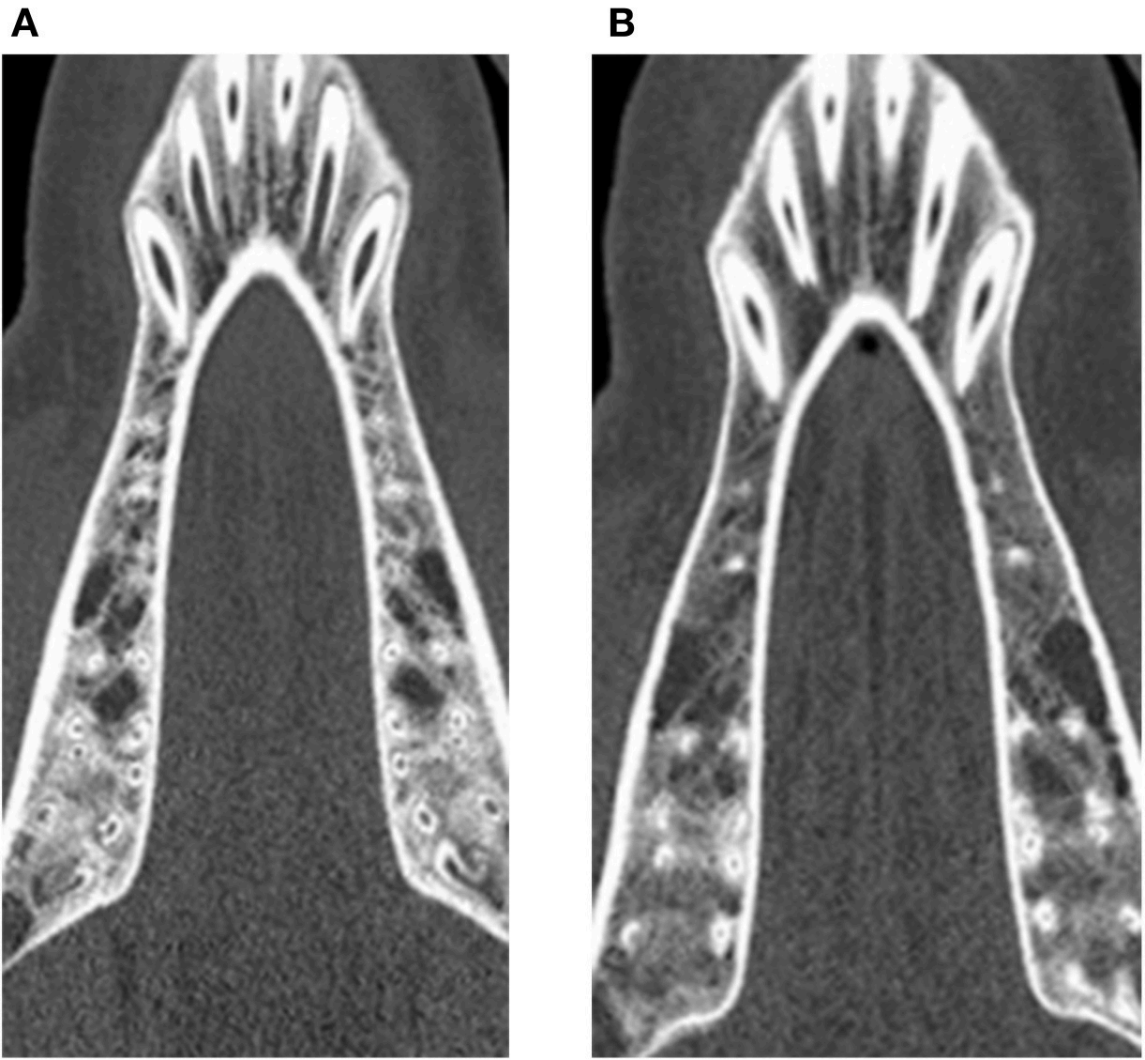
Trabecular rarefication over time shown in the mandible. Compared to the baseline **(A)** the spongious area in the region of interest appears in darker gray values after 9 months **(B)** indicating a loss of mineralization.

At the baseline point of the study, the values for BMD in the lumbar vertebrae and the maxilla differed statistically significantly between control and treatment group. For the mandible, no statistically significant difference could be observed (*p* = 0.973). In the computed tomography results after 6 months, the BMD showed a decrease in all evaluated regions. This decrease was statistically significant for both the control and the glucocorticoid group. Between the groups, no statistically significant differences could be found for any region. After 9 months, an increase of BMD in the control group was obvious for all evaluated regions compared to the 6-month values. This increase was statistically significant for all regions. However, the values after 9 months did not reach the baseline level again. Compared to baseline values, the decrease was statistically significant for the maxilla (*p* < 0.001). For the lumbar vertebrae and the mandible, the differences were not statistically significant (lumbar vertebrae: *p* = 0.981; mandible: *p* = 0.559). An example for the rarefied trabecular structure in the jaw bone is given in Figures 4A,B for the maxilla and in Figures 5A,B for the mandible.

After 9 months, there was an increase in the bone density values in the lumbar vertebrae in the glucocorticoid group compared to the 6-month value. This was statistically significant. In the jaw region, a slight decrease of BMD was observed. However, this decrease did not reach statistical significance when compared to the values after 6 months (maxilla: *p* = 0.551; mandible: *p* = 0.559). Compared to the baseline, the values after 9 months of glucocorticoid administration were lower. For the lumbar vertebrae region, this difference was not statistically significant (*p* = 0.981). In the jaw region, the difference reached statistical significance. When compared to the control group the values for BMD differed statistically significant for all examined regions in the glucocorticoid group. The detailed chronological data and statistical significant differences are shown in Table 1.

**Table 1:** Values of bone mineral density in Hounsfield Unit (HU).

**Region of interest**	**Baseline (HU)**	**6 months (HU)**	**9 months (HU)**
**Control**	**Mean ± SD**	**Mean ± SD**	**Mean ± SD**
Lumbal vertebrae	464.70^*1^ ± 46.44	382.71^*+3^ ± 40.90	444.67^+3,10^ ± 43.29
Maxilla	569.21^**^^$2,6^ ± 66.24	407.25^**#4^ ± 72.87	474.80^#$4,6,11^ ± 76.36
Mandible	613.41^″^ ± 74.99	537.14^″∧5^ ± 87.46	598.83^∧5,12^ ± 112.85
**GLUCOCORTICOID**			
Lumbal vertebrae	377.80^1^ ± 51.49	339.87^7^ ± 57.84	366.37^7,10^ ± 71.76
Maxilla	421.91^2,8^ ± 119.20	323.25 ± 69.74	315.51^8,11^ ± 71.23
Mandible	541.23^9^ ± 151.83	424.40 ± 106.84	402.89^9,12^ ± 99.23

Means and standard deviations (SD) are depicted. The statistical significant differences of the pairwise comparisons are marked (^*, **, +, $, 2, 3, 6, 8, 9, 11, 12:^ p < 0.001;”, ^#^, ^∧^, ^1, 4, 5, 7, 10:^ p < 0.05).

### Serum parameters

#### Control group

**Figure 6:**
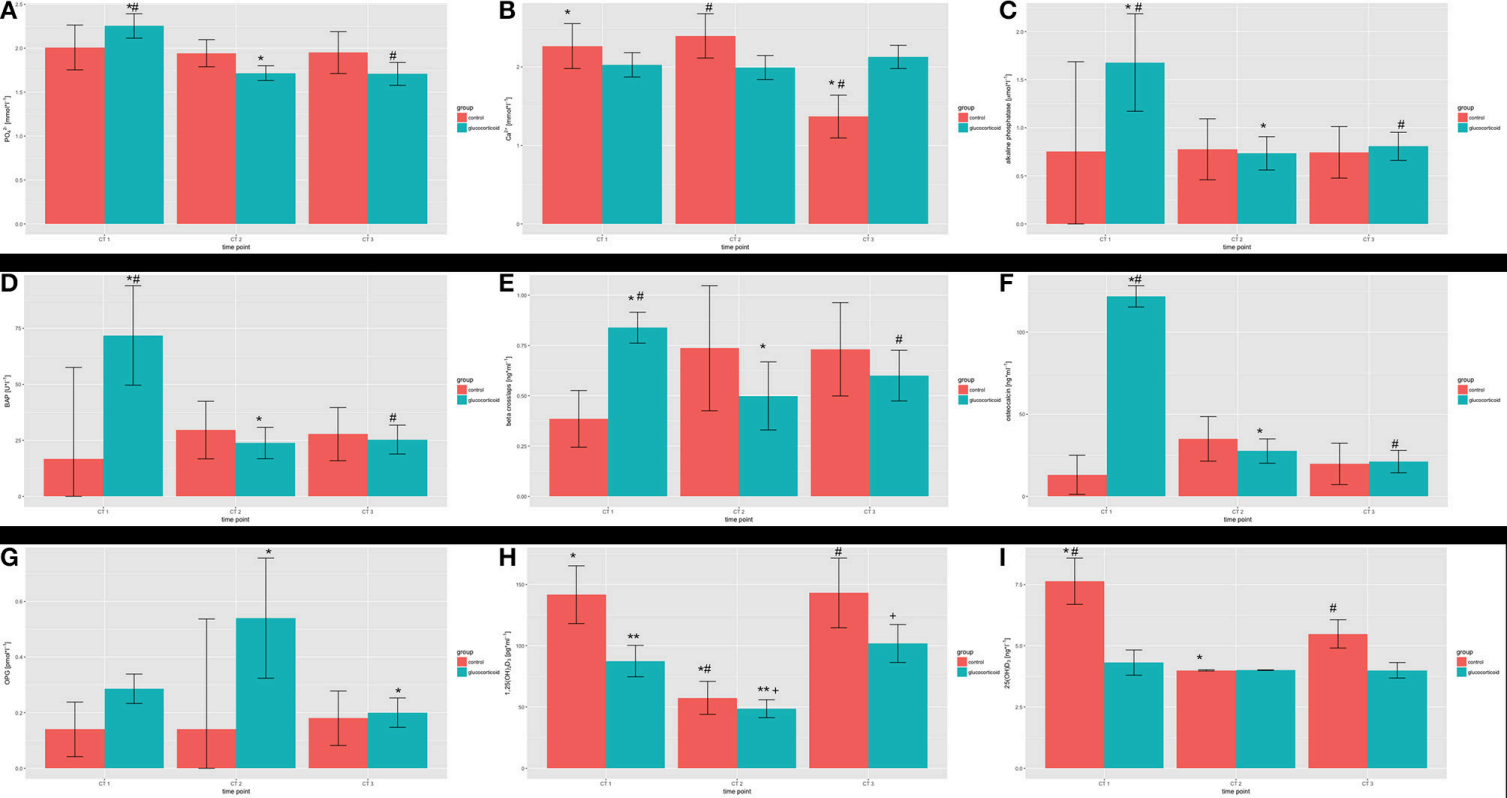
Serum parameters during the study period: **(A)** inorganic phosphorus; **(B)** calcium; **(C)** alkaline phosphatase; **(D)** bone specific alkaline phosphatase (BAP); **(E)** β-crosslaps; **(F)** osteocalcin; **(G)** osteoprotegrin; **(H)** 1,25-dihydroxyvitamin D; **(I)** 25(OH)-hydroxyvitamin D. Statistical significant differences within the groups over the study period are marked (^*^, ^**^, #, +).

In the control group, the level of ionic phosphorus decreased slightly during the period of study while values of ionic calcium, alkaline phosphatase, osteocalcin, and 1,25(OH)2-vitamin D varied. The values of bone specific alkaline phosphatase and beta crosslaps increased. During the study period, an increase could be observed for OPG serum levels. The means and their standard deviations are shown in Table 2. Statistical significant differences are depicted in Figure 6.

**Table 2:** The development of serum parameters over the study period.

		**CT 1**			**CT 2**			**CT 3**		
**Parameter**	**Group**	**Mean**	**SD**	**95% CI**	**Mean**	**SD**	**95% CI**	**Mean**	**SD**	**95% CI**
${\text{PO}}_{4}^{2-}$/mmol^*^l^−1^	Control	2.008	0.541	(1.752, 2.263)	1.943	0.188	(1.789, 2.096)	1.950	0.148	(1.712, 2.188)
	Glucocorticoid	2.253	0.285	(2.114, 2.392)	1.716	0.220	(1.633, 1.800)	1.708	0.365	(1.578, 1.838)
Ca^2+^/mmol^*^l^−1^	Control	2.266	0.440	(1.981, 2.552)	2.396	0.086	(2.115, 2.678)	1.368	0.381	(1.095, 1.640)
	Glucocorticoid	2.027	0.385	(1.872, 2.183)	1.993	0.439	(1.840, 2.147)	2.128	0.378	(1.979, 2.276)
AP/μmol^*^l^−1^	Control	0.754	0.161	(–0.178, 1.686)	0.776	0.222	(0.459, 1.093)	0.744	0.126	(0.476, 1.012)
	Glucocorticoid	1.678	1.457	(1.171, 2.185)	0.734	0.483	(0.561, 0.907)	0.807	0.415	(0.661, 0.953)
BAP/U^*^l^−1^	Control	16.738	4.194	(−24.081, 57.556)	29.588	5.192	(16.734, 42.441)	27.750	2.799	(15.887, 39.613)
	Glucocorticoid	71.830	63.894	(49.611, 94.048)	23.793	19.950	(16.796, 30.789)	25.330	18.523	(18.872, 31.787)
β-Crosslaps/ng^*^ml^−1^	Control	0.385	0.099	(0.244, 0.526)	0.736	0.172	(0.426, 1.047)	0.731	0.081	(0.499, 0.963)
	Glucocorticoid	0.838	0.215	(0.761, 0.915)	0.499	0.478	(0.330, 0.668)	0.600	0.361	(0.474, 0.726)
Osteocalcin/ng^*^ml^−1^	Control	13.125	3.605	(1.195, 25.055)	34.988	7.618	(21.428, 48.547)	19.763	8.833	(7.210, 32.315)
	Glucocorticoid	121.822	18.591	(115.328, 128.316)	27.563	20.865	(20.182, 34.944)	21.137	19.118	(14.304, 27.970)
OPG/pmol^*^l^−1^	Control	0.140	0	(0.042, 0.238)	0.140	0	(−0.257, 0.537)	0.180	0.091	(0.082, 0.278)
	Glucocorticoid	0.286	0.153	(0.233, 0.339)	0.540	0.622	(0.324, 0.756)	0.200	0.146	(0.147, 0.253)
1,25(OH)_2_D_3_/pg^*^ml^−1^	Control	141.625	27.184	(118.075, 165.175)	57.463	16.318	(44.053, 70.872)	143.050	51.234	(114.620, 171.480)
	Glucocorticoid	87.433	34.082	(74.614, 100.253)	48.633	19.220	(41.334, 55.932)	101.778	35.721	(86.302, 117.253)
25(OH)D_3_/ng^*^ml^−1^	Control	7.638	2.744	(6.692, 8.583)	4.000	0	(3.975, 4.025)	5.488	1.750	(4.908, 6.067)
	Glucocorticoid	4.315	0.407	(3.800, 4.829)	4.007	0.038	(3.994, 4.021)	4.000	0	(3.684, 4.316)

*Means, their 95% confidence intervals (CI) and standard deviations (SD) are depicted*.

#### Effects of glucocorticoid exposure on serum parameters

The serum values for ionic phosphorus showed a decrease over the study period. Ionic calcium values varied over the study period as in the control group. From the baseline to the measurement after 6 months, alkaline phosphatase and bone specific alkaline phosphatase decreased. After initiating the administration of glucocorticoids, the values for osteocalcin decreased after 6 months compared to the baseline. The serum levels for beta-crosslaps, OPG, and 1,25(OH)_2_-vitamin D varied. The detailed data is depicted in Table 2 and statistical significant differences are depicted in Figure 6.

#### Inter-group comparison

At baseline, the values for all serum parameters differed between the groups. A decrease could be observed for ionic phosphorus in both groups, but was more pronounced in the glucocorticoid group. The decrease differed statistically significant for both time points (*p* = 0.008; *p* = 0.009). When comparing bone-specific alkaline phosphatase, osteocalcin, and 25(OH)-vitamin D, the development in both groups differed significantly (alkaline phosphatase: *p* = 0.013; *p* = 0.017; osteocalcin: *p* < 0.001; 25(OH)-vitamin D: *p* < 0.001). No statistically significant differences were found for the trends of the levels of alkaline phosphatase (*p* = 0.076; *p* = 0.111), OPG (*p* = 0.266; *p* = 0.129). For 1,25(OH)_2_-vitamin D, the development initially differed between the groups (*p* = 0.006) but not for the entire study period (*p* = 0.443).

## Discussion

The osseointegration of dental implants is commonly analyzed in animal models prior to clinical application in humans. In treatment clinics however, the situation is often different to ideal pre-clinical laboratory conditions. Many patients suffer from local bone defects due to bone resorption following tooth extraction or as a consequence of local trauma and require bone augmentation prior to implant therapy. Further, an increasing number of patients suffer from systemic diseases such as diabetes mellitus or osteoporosis, and are receiving medical therapy. These factors might interfere with bone healing, osseointegration of implants, and the health of oral soft tissues (Sachelarie et al., 2016).

### Bone mineral density

In the present study, BMD was measured using QCT, which is frequently used in animal studies (Scholz-Ahrens et al., 2007; Veigel et al., 2011). The measurements are focused on the trabecular bone mineral density, as glucocorticoid administration seems to have an important effect (Wetzsteon et al., 2009; Paggiosi et al., 2015). Furthermore, the gray values of the cortical bone and the dental structures are in the same range. Thus, cortical bone has usually been excluded from the measurements to avoid a biased analysis through inclusion of dental structures (roots). Scholz-Ahrens et al. found a statistically significant decrease in BMD in lumbar vertebrae after 8 months of glucocorticoid treatment (Scholz-Ahrens et al., 2007). This partially agrees with the results of this current study, which showed a statistically significant decrease in lumbar vertebrae after 6 months with a slight increase of BMD after 9 months. This might be related to an increase of body weight which is described for minipigs after completed adolescence (Richel and Waldmann, 2015). The growing weight might be a stimulus for bone formation in the vertebral region due to an increase of stress. An unexpected finding in the current study were the statistically significant differences considering the baseline values of bone mineral density. One possible reason for the different baseline data might be inter-animal variance. All came from the same herd, however, showed a variance in age and weight. Another reason might be the different group size (8 vs. 27 animals completing the study). If the control group would have had an equal group size to the glucocorticoid group, the differences might have been lower due to the compensation of extremal values.

The results for maxillofacial BMD in animals undergoing the induction of osteoporosis are different to those of lumbar bone. In a study using ovariectomized sheep, a pronounced horizontal and vertical bone loss in the mandible was described after 3 and 12 months (Johnson et al., 1997). However, a metric measurement of bone volume was not performed. Using the same animal model, another study measured BMD 12 months after ovariectomy, and showed no changes in the region of the diastema or the mandibular ramus (Johnson et al., 2002). Bone density in the alveolar regions however was significantly decreased, by 27.8%. This is in accordance with our results, which showed a progressive rarefication of the trabecular structure of the maxilla and the mandible. This rarefication of trabecular bone was found to be more pronounced in mandibular bone. In the experimental group, both a progressive decrease over time and a decrease in comparison to the control group were recorded.

### Animal models

The consideration of bone quality raises the question of appropriate animal models to simulate human osteoporosis. Osteoporotic large animal models have been established in sheep, dog, and pigs (Volozhin et al., 1990; Scholz-Ahrens et al., 2007; Dvorak et al., 2011b; Zhang et al., 2014; Kielbowicz et al., 2015). The most frequently described large animal model seems to be the sheep (Dvorak et al., 2011b; Veigel et al., 2011; Kielbowicz et al., 2015). With regard to the oral and maxillofacial situation, the minipig seems to be the most suitable model. First, it is an omnivore, so the diet has more similarities to a human diet than do sheep and dog models. Furthermore, the bone and oral tissues structure as well as bone turnover is quite similar to humans (Hönig and Merten, 1993; Hönig et al., 1997; Wang et al., 2007; Swindle et al., 2012). In a review, Pearce et al. concluded that both bone composition and bone remodeling in pigs are largely similar to that of humans, while micro- and macrostructure can be considered as moderately similar (Pearce et al., 2007). Regarding the age, the minipigs included in the current study can be considered as young adults. The life span of the breed Mini-Lewe was described with 10–15 years (Leucht et al., 1982). Richel observed in a longitudinal study of 144 Mini-Lewe pigs no further increase in the withers height and crown rump length after an average age of 20 months. Day 600 post-partum was defined as the time point of completed adolescence (Richel and Waldmann, 2015). This is in accordance with the findings of the current study, as no bone growth could be determined when comparing the matched images of the CT scans during the study period. Unlike in humans, in non-primate mammals there is no description of a physiological menopause state in the literature (Finn, 2001). Therefore, the use of young adult minipigs seems to be appropriate as there is no change in estrous cycle expected with increasing age. Additionally, when implant osseointegration is evaluated, the anatomy of the jaw of minipigs allows the application of the same instruments as used for human patients (Stadlinger et al., 2012).

### Serum parameters

Any systemic animal model needs to be validated by systemic parameters. Along with the measurement of bone density, the analysis of serum parameters is of particular interest, providing better understanding of bone metabolism.

#### Inorganic phosphorus

The values of the inorganic phosphorus decreased in both groups which was more pronounced in the glucocorticoid group. When comparing the current values to the literature the values of the control group were in the same range (2.38 ± 0.86 to 3.10 ± 0.65 mmol^*^l^−1^; Mieth, 1978; Leucht et al., 1982; Gusewski, 1983; Richel and Waldmann, 2015). Increasing age was discussed as a possible reason for this decrease by Gregor et al. but could not be confirmed in other evaluations (Gregor, 1979; Richel and Waldmann, 2015). In a recent study, inorganic phosphorus values between 1.55 and 2.24 mmol^*^l^−1^ were measured in female Aachen minipigs, a breed descending from the Mini-Lewe strain (Pawlowsky et al., 2017). However, these differences might be due to a different state of growths as the used animals were aged 6 months. When comparing both groups during the study, lower phosphorus levels were obvious in the glucocorticoid group. This state might be considered as hypophosphatemia which possibly is caused by the reduced intake of vitamin D3 of the glucocorticoid group. Furthermore, hypophosphatemia could be corresponding with lower levels of 1,25(OH)2-vitamin D3 in the glucocorticoid group what could be observed in the present study. One possible reason for the lower phosphorus values might be an interaction of glucocorticoids and the phosphate balance (Levi et al., 1995).

#### Calcium

The values of serum calcium varied over the study period in both groups ranging from 1.368 to 2.396 mmol^*^l^−1^. Values <2.1 mmol^*^l^−1^ in humans are considered as hypocalcemia (Fong and Khan, 2012). In the literature, values between 3.1 and 2.5 mmol^*^l^−1^ are stated (Mieth, 1978; Leucht et al., 1982; Gusewski, 1983; Richel and Waldmann, 2015). Symptoms of acute decreased serum calcium levels include muscle spasms, heart failure, and neuromuscular irritability whereas a slow decrease of calcium levels might remain without symptoms (Fong and Khan, 2012). As no of the animals in the present study developed comprehensibly any of these symptoms it is unlikely that there was an apparent hypocalcemia.

#### Alkaline phosphatase and bone-specific alkaline phosphatase

The values of total alkaline phosphatase and bone-specific alkaline phosphatase showed a significant decrease after glucocorticoid administration for 6 months. These findings were consistent with those of Ikeda et al., who observed a reduction of bone-specific alkaline phosphatase and osteocalcin values in serum of adolescent miniature pigs under glucocorticoid treatment (Ikeda et al., 2003). This might be explained by decreased bone formation due to supressed osteoblast activity (Lukert et al., 1986; Prummel et al., 1991). Clinically, a statistically significant correlation between bone alkaline phosphatase as well as osteocalcin levels and the age was found in postmenopausal women (Lumachi et al., 2009). However, a correlation between these bone markers and the BMD was not observed.

#### β-crosslaps

The C-terminal telopeptide of the type I-collagen is termed as β-Crosslaps. It is considered as bone resorption marker (Okabe et al., 2004). In a clinical study, the changes of β-Crosslaps in serum correlated with the annual changes in BMD in postmenopausal women (Christgau et al., 1998). They are of crucial value to monitor the effect of antiresorptive therapies (Christgau et al., 1998). Furthermore, an increased level of β-Crosslaps and OPG was observed in patients suffering from Wilsons disease and osteoporosis (Hegedus et al., 2002). No such results could be observed in the present study. A reason might be a wide interindividual variety of the serum levels. However, there is little literature describing the levels of β-Crosslaps in pigs.

#### Osteocalcin

The osteocalcin serum levels in the glucocorticoid group decreased significantly after 6 months of prednisolone administrations. This was not observed in the control group. In human studies, osteocalcin was described as a sensitive marker for osteoblastic depression under glucocorticoid therapy (Lukert et al., 1986; Ekenstam et al., 1988). The effects were pronounced when doses of glucocorticoids between 5 and 30 mg were administered daily over a period of at least 3 months (Lukert et al., 1986). In our study, a daily average dosage correlating to 35–45 mg prednisolone in human subjects (based on body weight) over a period of more than 4 months was assumed. This implies a comparable effect on the osteocalcin level (Scholz-Ahrens et al., 2007). However, the baseline values of both groups differed significantly, limiting this comparison. One possible reason for low values of osteocalcin might have been the degradation of osteocalcin by proteases of erythrocytes and leukocytes. In cases of difficult blood drawing degradation might have started already before further processing of the blood samples.

#### OPG

OPG is a glycoprotein that regulates bone density (Yano et al., 1999). A significant correlation was found between OPG serum level and age (Yano et al., 1999; Fahrleitner-Pammer et al., 2003). There was no such finding in the present study. Possibly, the age range of the minipigs was too small for the observation of such an effect. In contrast to our study, where young adult animals were examined the mentioned study evaluated patients with an age ranging from young adults to elderly patients. Furthermore, there is an increased level of OPG in patients suffering from osteoporosis (Yano et al., 1999). In the present study, slightly increased levels of OPG were observed in the glucocorticoid group during the study period. This, however, showed no significant statistical difference when compared to the control group. In a study of ovariectomized rats, a transient increase of OPG was found in the initial stage of osteoporosis, which might correlate with the increase of OPG observed in our study (Miyazaki et al., 2004).

#### 1,25-dihydroxyvitamin D and 25(OH)-hydroxyvitamin D

The levels measured for 1,25-dihydroxyvitamin D were variable over the study period in both groups. This is in accordance to the literature, where increases, decreases, and an absence of alterations of the 1,25-dihydroxyvitamin D levels in individuals undergoing glucocorticoid therapy are described (Hodsman et al., 1991; Prummel et al., 1991; van der Veen and Bijlsma, 1992). However, in contrast to our study, higher glucocorticoid doses given intravenously cause a pronounced decrease (van der Veen and Bijlsma, 1992). To minimize extraneous influences, animals were housed in a stable and both groups received an equally vitamin D3 deprived (<300 IU/kg) daily diet. Comparable to other studies, no strong correlation could be found between the changes in bone metabolism and serum levels of 1,25-dihydroxyvitamin D (Seeman et al., 1980).

In summary, the serum parameters in the current study showed a wide range of values. In comparison to the literature, there are concordances but also some discrepancies. One potential reason is the fact, that different breeds of minipigs are being compared. Furthermore, in contrast to humans, there are no standard values for most parameters in minipigs. For some parameters, there were no reference values of pigs available at all. Thus, only a comparison to values obtained from other species was possible. Here, further studies are needed to elucidate potential correlations. Another possible reason might be the use of different analytical methods (Richel and Waldmann, 2015).

### Methods of osteoporosis induction

The induction of an osteoporotic state in large animal models has been described by means of ovariectomy, calcium-deficient diet, glucocorticoid administration, and combinations of these approaches (Scholz-Ahrens et al., 1996, 2007; Kielbowicz et al., 2015). In comparison to deficient nutrition and glucocorticoid application, ovariectomy carries the disadvantage of a surgical intervention, which is a cause of stress for the animals. Furthermore, the effect of ovariectomy on nulliparous animals has been reported to be limited (Scholz-Ahrens et al., 1996). This would apply to the animals in the current study. Additionally, ovariectomy creates a sudden change in the hormonal balance, which is not entirely comparable to the postmenopausal situation, where the hormone levels change more gradually. In distinction, the administration of glucocorticoids over a period of time might lead to a gradual induction of osteoporosis, more closely mimicking the natural disease condition. However, a longer period of glucocorticoid administration may be required to show measurable effects on bone density. One limitation of this study might be the unequal size of the animal groups. This might cause some bias in the statistical evaluation e.g., statistically significant differences in BMD at the baseline. The reason was to minimize the total number of animals used. This was similar to other studies, where unequal group sizes for treatment and control groups have also been used (Scholz-Ahrens et al., 2007; Kielbowicz et al., 2015).

Compared to intramuscular application of glucocorticoids one limitation of oral administration is the fact that the individual animal dose is not fully calculable, because of the uncertainty about complete ingestion and absorption. Furthermore, glucocorticoids have various metabolic effects not limited to the stimulation of bone resorption and induction of osteoblast apoptosis (Dalle et al., 2001; Spreafico et al., 2008). Regarding the suitability as a model for dental implant research, glucocorticoid administration might lead to delayed wound healing. Morin and Fardet described altered wound healing in a retrospective study in humans, when glucocorticoids were taken for more than 6 months (Morin and Fardet, 2015). Dental implant clinics are seeing an increasing number of patients with progressive osteoporosis secondary to the use of glucocorticoid medications for the treatment of rheumatoid arthritis or following organ transplantation. Thus, despite possible criticisms of the animal model, glucocorticoid-induced osteoporosis might be a fair representation of a common clinical situation.

## Conclusion

There are advantages and disadvantages of every animal model which involves artificial induction of a compromised metabolic state. However, the induction of an osteopenic state in the minipig by oral administration of glucocorticoids is predictable, and is minimally invasive when compared to surgical ovariectomy. In summary, the induction of osteoporosis in the minipig appears to be a promising model for the evaluation of the osseointegration of dental implants under systemically impaired osseous conditions.

## Author contributions

MS was involved in the data acquisition, analysis and interpretation, drafted the manuscript, approved the final version of the manuscript, and agreed to be accountable for integrity and accuracy of the work. JK, SE, and RJ were involved in the data acquisition, drafted the manuscript, approved the final version of the manuscript, and agreed to be accountable for integrity and accuracy of the work. EK and LH were involved in data analysis and interpretation, drafted the manuscript, approved the final version of the manuscript, and agreed to be accountable for integrity and accuracy of the work. RM, UE, and CS were involved in study conception, drafted the manuscript, approved the final version of the manuscript, and agreed to be accountable for integrity and accuracy of the work. BS was involved in the conception of the study, data interpretation, drafted the manuscript, approved the final version of the manuscript, and agreed to be accountable for integrity and accuracy of the work.

### Conflict of interest statement

The study was supported by Dentsply Sirona Implants, Mannheim, Germany. LH was supported by the Transregio 67 subproject B2 of the German Research Foundation DFG (Deutsche Forschungsgemeinschaft). The other authors declare that the research was conducted in the absence of any commercial or financial relationships that could be construed as a potential conflict of interest.
